# Why Don’t Smokers Want Help to Quit? A Qualitative Study of Smokers’ Attitudes towards Assisted *vs.* Unassisted Quitting

**DOI:** 10.3390/ijerph120606591

**Published:** 2015-06-10

**Authors:** Kylie Morphett, Brad Partridge, Coral Gartner, Adrian Carter, Wayne Hall

**Affiliations:** 1 University of Queensland Centre for Clinical Research, Royal Brisbane and Women’s Hospital Site, Herston, 4029 Queensland, Australia; E-Mails: c.gartner@uq.edu.au (C.G.); adrian.carter@monash.edu (A.C.); w.hall@uq.edu.au (W.H.); 2 University of Queensland, School of Public Health, Corner of Wyndham Street and Herston Road, Herston, 4006 Queensland, Australia; 3 University of Queensland Centre for Youth Substance Abuse, Mental Health Centre, Royal Brisbane and Women’s Hospital, Herston, 4029 Queensland, Australia; E-Mail: bradparto@hotmail.com; 4 School of Psychological Sciences, Monash University Clinical and Imaging Neuroscience, 770 Blackburn Road, Monash University, Clayton, 3800 Victoria, Australia

**Keywords:** smoking, smoking cessation, medicalization, attitude, qualitative research

## Abstract

The development of prescription medication for smoking cessation and the introduction of evidence-based guidelines for health professionals has increasingly medicalised smoking cessation. There are debates about whether medicalisation is a positive development, or whether it has devalued unassisted quitting. In this debate the views of smokers have been neglected. This study explored the attitudes of smokers towards a range of quitting methods, and their considerations when judging their value. We conducted semi-structured interviews with 29 smokers and analysed data using thematic analysis. The results show that the perceived nature of an individual smoker’s addiction was central to judgments about the value of pharmacological cessation aids, as was personal experience with a method, and how well it was judged to align with an individual’s situation and personality. Unassisted quitting was often described as the best method. Negative views of pharmacological cessation aids were frequently expressed, particularly concerns about side effects from prescription medications. Smokers’ views about the value of different methods were not independent: attitudes about cessation aids were shaped by positive attitudes towards unassisted quitting. Examining smokers’ attitudes towards either assisted or unassisted quitting in isolation provides incomplete information on quitting preferences.

## 1. Introduction

Smoking cessation has become increasingly medicalised since the introduction of nicotine replacement therapy (NRT) in the 1970s. More recently, increased knowledge about the physiological mechanisms of nicotine dependence have led to the development of new medications, such as varenicline, which increase the chances of successful cessation [1,2]. Clinical guidelines now encourage health professionals to identify smokers and facilitate quit attempts by prescribing pharmacological cessation aids and/or referring smokers to counselling services [3,4,5]. A recent commentary has called for treatment to be provided to all smokers who attend a health provider, not just those who express readiness, or an interest in quitting [6].

A number of commentators have been critical of this medicalised approach to smoking cessation. Some have concluded, based on population-based observational studies, that pharmacological cessation aids are no more effective than no treatment [7]. Others have argued that while pharmacological treatments have demonstrated efficacy in clinical trials, these individually focused treatments have not significantly reduced smoking prevalence [8,9]. It has also been suggested that the promotion of cessation aids by pharmaceutical companies may imply that quitting without formal assistance is more difficult than it is, thereby undermining smokers’ willingness to try to quit and their belief in their ability to stop [8,10,11]. Chapman and McKenzie argue that unassisted quitting, or “cold turkey”, has the greatest impact on reducing smoking prevalence and accordingly should receive greater clinical and research attention [10].

This debate has implications for public health messages about how smokers should quit, and how smoking cessation is discussed in clinical interactions. Should people who smoke be told, as has been recently suggested, that they have a “chronic, relapsing disease”? [12]. Should they be informed that it is difficult to quit and relapse is likely? Do we want them to believe that smoking cessation requires treatment by a health professional? Or should they be informed that the majority of people quit unaided; that quitting is often easier than anticipated; and that with motivation and willpower, they can quit on their own?

The aim of this paper is not to conclusively answer these questions, but to explore how smokers themselves evaluate, and deliberate on, different methods for quitting smoking. Public health researchers have long recognised and examined the influence of lay beliefs about health and illness on health-related behaviours such as treatment choice and adherence [13,14,15]. For example, Horne and Weinman [16] found that treatment adherence was predicted by the difference between beliefs about the necessity of treatment and concerns about side effects. Research conducted with smokers on their beliefs about smoking cessation has typically surveyed their attitudes towards specific quitting methods, usually with the aim of identifying barriers to the use of pharmacological cessation aids. These studies have shown that smokers often display negative attitudes towards pharmacologically assisted cessation and express concern about their safety and efficacy [17,18,19]. Not surprisingly, these negative perceptions predict a lower intention to use pharmacotherapies and poorer adherence in those smokers who do use them [20,21,22,23].

Quantitative research on perceptions of safety and efficacy may provide an incomplete view of the factors that smokers consider when making choices about how to quit. In addition, few of these studies have examined views on the most common method that people use to quit: “cold turkey” or quitting unassisted. A systematic review of the Australian literature on how smokers quit found that only 19 of 185 studies included data on unassisted quitting [9]. A recent study that did include data on unassisted quitting found that NRT and prescription medications were rated as helpful by those who had used them. However, unassisted quitting was used substantially more often than either, and also rated as helpful [24].

Qualitative research may provide a more nuanced account of smokers’ attitudes towards treatment for smoking. For example, smokers have reported that NRT did not reduce their cravings and that they were concerned about becoming addicted to it [25,26]. Additionally, NRT was not seen to address the critical role of willpower in quitting smoking, or the ways in which cigarette smoking was intertwined with routine and social aspects of everyday life [27]. Many young smokers felt they did not require NRT because they did not see themselves as physiologically addicted [28].

While the existing qualitative research has provided insight into smokers’ thoughts about quitting in general, there is little that has compared smokers’ views about multiple different quitting methods. This paper addressed this gap by providing a nuanced view of (1) smokers’ attitudes towards assisted and unassisted quitting; and (2) the factors that smokers take into account when evaluating and comparing different methods of quitting.

## 2. Methods

Semi-structured interviews were conducted with 29 daily smokers aged 18 years or over from a large metropolitan Australian city. We employed purposive sampling in order to document the breadth of ideas about quitting methods. Prior to the interview commencing, a short survey with questions about demographics and smoking history was completed by participants (see Supplementary file). The recruitment strategy was periodically adjusted as required to obtain a “maximum diversity” sample in relation to age, sex, education, and socioeconomic status [29]. For example, flyers were distributed to neighbourhood community centres in order to recruit socially disadvantaged smokers. A university mailing list was employed to recruit university-educated smokers, and a seniors database used to recruit older participants. Other methods included handing out flyers in person, advertising via an online classified site, and placing the adverts on community noticeboards. We judged that thematic saturation had been reached at 29 interviews, when a sufficiently diverse sample had been obtained and no new themes were emerging from ongoing analysis. Participants were provided with a gift voucher in appreciation for their time. All recruiting and interviewing was conducted by Kylie Morphett (KM) between October 2012 and July 2013.

The interview questions reported here are a subset from a larger project about neurobiological understandings of nicotine addiction. Participants were asked about their attitudes toward various methods for quitting smoking, specifically: “*What is your view on the following methods for people trying to quit?*” This initial exploratory question was designed to elicit unprompted views about the methods, in order to ensure a space for emergent themes. Prompts were then provided where appropriate. Example prompts were: “*Do you have any experience using (insert method)? Do you think it is safe? Do you think it is effective?*” Participants were asked what method they would choose if they were to make a quit attempt and why; and were asked to describe any previous quit attempts.

All participants were asked about each of the following methods for quitting: (1) no treatment (prompt: cold turkey); (2) nicotine replacement therapy (prompt: gum, patches); (3) prescription medication (prompt: Champix, Zyban); (4) counselling, including the *Quitline*; and (5) self-help materials (prompt: books, information pamphlets). We also asked whether participants had thoughts on any other methods not mentioned.

Interviews were recorded and transcribed verbatim. They ranged in length from 25 min to one hour and 20 min. The confidentiality and anonymity of participants was maintained at all times through adherence to standard ethical procedures [30]. All participants provided informed consent and the study was approved by the Human Research Ethics Committee of The University of Queensland (Project Number: 2009001022).

We employed thematic analysis, as described by Braun and Clarke, to analyse the data [31]. Thematic analysis has been described as the most useful method for “capturing the complexities of meaning within a textual data set” [32] andit is well suited to exploratory studies using interview data. An inductive approach was utilized, whereby KM developed descriptive codes based on patterns observed in the data and conducted a critical analysis of these codes in order to collate them into major themes. Data coding was conducted using NVivo 10 software [33]. Another author (Brad Partridge) read the transcripts and developed themes independently. There was good agreement about the themes and any discrepancies were discussed until a consensus was reached. In addition, another member of the research team conducted double-coding of a subset of data in NVivo in order to ensure that the final coding scheme had adequate reliability.

## 3. Results

### 3.1. Participants

Participant demographics are presented in Table 1.

In order to gain an overview of participant experience with quitting methods, basic data was collected in the pre-interview survey about quitting history. Participants were presented with a list of methods adapted from the Australian National Drug Strategy Household Survey [34] and asked to select the methods that they had used. Multiple selections were permitted. Four participants reported that they had not previously made a quit attempt. The quitting strategies that the remaining participants reported having used are listed in Table 2. Though NRT was the method that the most participants reported having used, a significant proportion of the participants had no direct experience with pharmacological cessation aids.

**Table 1:** Participant demographics (*n* = 29).

Demographic	Number
Gender	
Male	15
Female	14
Age (years)	
18–25	9
26–40	11
41–54	4
55+	5
Highest level of education	
No formal qualification	4
Secondary school	4
Post-secondary qualifications (e.g., trade training)	10
University degree	11
Employment status *	
Employed	15
Unemployed	5
Student	7
Retired/Pensioner	3
Cigarettes per day **	
1–10	10
11–20	11
21–30	3
31+	4

* Multiple selections permitted; ** Missing data = 1.

**Table 2:** Strategies used on previous quit attempts.

Method	Used
Discussed smoking and health at home	10
Contacted the “QUIT” line	3
Asked your doctor to help you stop smoking	4
Used nicotine gum, nicotine patch, or nicotine inhaler	11
Used a smoking cessation pill (e.g., Zyban, Champix)	3
Bought a product other than nicotine patch, gum or pill	2
Read “How to Quit” literature	9
Used the internet to help you quit	5
Done something else to help you quit?	7
None of the above	8

### 3.2. Unassisted Quitting

Unassisted quitting was frequently described as the best way to quit smoking and it was participants’ overwhelmingly preferred method for their next quit attempt. A number of justifications were provided for this preference. First, there was a belief that if someone had a strong desire to quit and was “ready” for it, then assistance would not be required. Desire and motivation was seen as the foundation of quitting success:

*You’ve really got to want to do it and have that courage, strength, determination to do it. You’ve really got to have that thinking in your mind, this is what I want. I personally believe that the mind has a most powerful part in this whole process*.

(*Female 55+, 21–30 cigarettes per day (CPD)*)

This was tied to the belief that cold turkey would only be effective at a time when someone had reached a point where they “really wanted” to quit. Many of our participants said that willpower, or a strong desire to quit, was a necessary condition for a successful quit attempt. However this seemed to imply that that if a person failed in their attempt to quit cold turkey, then this meant that they hadn’t really wanted to quit; that they weren’t strong enough; or that they didn’t have the right “mindset”. For example, the participant quoted below had stopped smoking and relapsed a number of weeks later. She attributes this relapse to not “wanting” it enough.

Because I always imagined if you’d stopped for a few weeks, how would you go about having that first cigarette? You would be just like no it's not worth it, but I did. I don’t even know when it was. It was probably I was out with my friend and I didn’t even realise I did it. You know I just-yes. I think if you want, I think that’s the main way. If you don’t want to do it, you’re not going to do it.

*(Female, 18–25, 1–10 CPD)*

Another participant implied that his failed quit attempts were due to a lack of internal strength or desire.

It depends how strong you are and how much you want to. If you are strong, if you really want it, you know. I couldn’t do it. Simple as that, I’m still smoking.

*(Male, 41–54, 31+ CPD)*

High value was placed on the sense of achievement that was anticipated as a result of quitting unassisted. This was more common amongst younger male participants, who saw quitting smoking as a challenge or a competition with oneself. Seeking assistance in the form of other cessation aids was seen as a “crutch” or a form of “cheating” that would mean you had not won against smoking:

I think it would be more of a trial for myself. Like a goal setting thing. I’m a very goal-orientated person. If I can go cold turkey that would be like a big achievement for me.

*(Male, 18–25, 1–10 CPD)*

The role of personal experience was a salient consideration when participants spoke about unassisted quitting. Most participants had tried to quit cold turkey, so could reflect on their own experience with this strategy of smoking cessation. As described above, some attributed past failures to personal weakness or a lack of desire to quit. There were other participants who considered past unassisted quit attempts successful, despite the fact that they had relapsed and were still smoking.

*Interviewee:* *I’ll just determine that I want to quit and I can.*

*Facilitator:* *Why would you use that method now?*

*Interviewee:* *Because I’ve tried it before and it’s working for me. Yeah, so I think that’s the easiest one.*

*(Male, 26–40, 1–10 CPD)*

The experiences of friends and family were sometimes used to justify an inclination or disinclination to use unassisted quitting.

Terrified. I know people who’ve done it but they’ve usually gone back to smoking again. They’ve often really struggled. I’ve seen people be very stressed and distressed during the cold turkey. So clearly some are able to do it but it looks pretty difficult.

*(Female, 55+, 11–20 CPD)*

Despite many stating a preference for quitting cold turkey, it was common to acknowledge the difficulties associated with it. Indeed, cold turkey was sometimes seen as both the hardest and best way to quit. The major difficulties were attributed to withdrawal symptoms and cravings. Some smokers, particularly those with a history of failed cold turkey quit attempts, thought that the method was better suited to those who were stronger or who had more willpower than themselves.

It depends on the person. I mean some people can do that and some people have the willpower or the determination to do it. They don’t need aids but yeah most people would.

*(Female, 26–40, 31+ CPD)*

The perceived level of addiction was another factor participants saw as important for unassisted quitting. Unassisted quitting was seen as most suitable for those who were not heavily addicted to cigarettes. The participant below equates heavy smoking with dependence.

I have friends who quit like that, cold turkey, and it worked out pretty well. But then again, they’re not those really heavy ones so I guess it works for people like us who aren’t that hooked on that shit yet.

*(Male, 18–24, 1–10 CPD).*

### 3.3. Assisted Cessation

Nicotine replacement therapy is the most commonly used pharmacotherapy for smoking cessation. In Australia, the cost of NRT is heavily subsidised if participants attend their doctor and receive a prescription. Despite this, only four of 29 participants intended to use NRT on their next quit attempt. Cost was mentioned as a barrier to the use of NRT by some participants, which could indicate a lack of awareness about government subsidization. However, a more common consideration was the individual’s assumption about the nature of a smoker’s addiction. NRT was thought to be most appropriate for those with a “physical” or “physiological” addiction. Many described themselves as addicted to the act of smoking and saw their addiction as “psychological”, or as a habit built into their daily routine. These participants did not necessarily have negative views of NRT; but thought it was more suitable for smokers who had a “real” physiological addiction.

(NRT) might be extremely effective on people who are very physically addicted. If they’re psychologically addicted I don’t see how it’s going to have any effect.

*(Female, 41–54, 11–20 CPD)*

NRT was seen by some of these smokers as failing to deal with the psychological or routine aspects of smoking that they considered central to their dependence.

I think it could help some people, but still it’s because it’s such a habit to smoke it’s not just the nicotine. ... Each cigarette we smoke is the fact of doing it, is having the pack in your bag, it’s like all those things should be replaced and so probably replacing it could help the craving for those people who are very hooked up. But I don’t think it would completely solve the issue and it wouldn’t definitely help 100% to quit smoking, there are a lot of other things involved.

*(Female, 18–25, 1–10 CPD)*

A small number of participants who had experience using NRT acknowledged the role of physiological dependence in their smoking and thought that NRT had been effective for them because it dealt with the physiological aspect of smoking. It allowed “breathing space” to deal with the more habitual, routine aspects of smoking.

Yeah, I think that does help because it does take away that initial physical withdrawal feeling so that you can concentrate on trying to manage the habit part of it. That, for me as I said, it only took a couple of weeks for me to get that clearing out of my system and then it was just a matter of trying to manage the ritual habit part of it. So that definitely made it a lot easier.

*(Female, 26–40, 31+ CPD)*

As with unassisted cessation, an individuals’ experience with NRT played a key role in their attitudes towards it. Participants rarely reported using NRT as directed. Rather, participants were more likely to use NRT short-term during long-haul flights or short-term stays in hospital. It was also used by a small number of participants as a one-off “experiment” to see what would happen:

The patches-we’ve got the patches on and we’ve just-we’ll see if that works. We’re not trying to give up smoking. I’ve just left them on there and thought, right I’ll have a cigarette when I want a cigarette.

*(Male, 41–54, 31+ CPD)*

Personal experience was particularly salient in relation to side effects. Those who had used NRT and experienced unpleasant side effects reported that they would not use it again. Even hearing about someone else’s experience of side effects was enough to dissuade people from using NRT:

I’m kind of skeptical on all the other stuff-the products on the market to stop it, patches and stuff like that. I’m kind of-I don’t know. Because I had a friend who used the patches and he used to have nightmares and-yeah, stuff like that. So I’m not too keen on it.

*(Male, 18–25, 1–10 CPD)*

A small number of participants were concerned about developing dependence on NRT. They saw dependence on nicotine as a negative state, with there being no essential difference between whether they consumed nicotine via smoking cigarettes or via NRT.

At some point you do need to just stop. You can’t just keep feeding your body this drug that you’re addicted to, you might as well be smoking.

*(Female, 26–40, 11–20 CPD)*

The prescription medications bupropion and varenicline are publicly subsidised forms of pharmacotherapy for smoking cessation in Australia. Approximately one third of participants were unaware of the existence of these prescription medications for smoking cessation. Because direct to consumer advertising of prescription medications is not permitted in Australia, this is perhaps not surprising. Amongst our participants, those who were older and heavier smokers were more likely to be aware of these medications. Those who did know of these medications frequently expressed concern about their safety. Cost was mentioned less often, perhaps because the fear of side effects dominated considerations of costs. While only a few had tried prescription medications for smoking cessation, many had heard reports about adverse side effects from their friends, family or acquaintances. The most commonly mentioned were mental health issues and nightmares. These side effects were cited as the main reason why most would not consider using prescription medication.

Then they try and tell me that these medications will stop me smoking although I’ll have nightmares, I’ll have all the other side effects. I heard about one, I can’t remember the name of it, and my dad had it-reckoned he nearly died. Made him really sick. I’ve heard about people having the nightmares and things, so that really makes me question what they’re giving you other than nicotine. You might stop smoking, but you’re just as irritable from not sleeping. So, I don’t know. I just don’t agree with the pharmaceuticals. If you’re going to quit, quit.

*(Male, 26–40, 11–20 CPD)*

This dislike of prescription medications for smoking cessation was sometimes an expression of a more general dislike of “relying” on any sort of medication. The participant quoted below positioned “taking pills” as an extreme measure for smoking cessation, especially when quitting without assistance was a realistic alternative. It may be that a “reliance” on medication conflicts with the value of self-reliance that many participants identified with.

I’m really against, not against, but I think like taking pills and taking things like that should be done only if it’s really needed and as long as I feel like I could do it without, it would always be better option than relying on medicine.

*(Female, 18–25, 1–10 CPD)*

As with NRT, some participants thought that prescription medication would be more suitable for “other” smokers with a more serious addiction; they were not inclined to use these medicines themselves. Medications were associated with “illness” and “sickness” that heavier and older smokers might develop.

I personally just can’t get my head around doing something like a pharmaceutical pill or something like that … It seems over the top but I understand that some people who are really ill and continue smoking will probably need that.

*(Male, 26–40, 11–20 CPD)*

As with NRT, perceptions of efficacy were also closely tied to the experiences of family and friends who had used these medications.

…Some of my friends have tried both of those and I still find that they’re smoking so I’ve seriously questioned that. Maybe their commitment wasn’t strong enough or whatever. But yeah I’m just still hoping for the wonder drug to be out there or something.

*(Female, 55+, 11–20 CPD)*

As the quote demonstrates, having sufficient willpower was still perceived as important, even when medication was taken. The participant expressed a hope that a “wonder drug” would be developed that, presumably, would overcome this need for willpower or sustained effort. However, medication was not generally seen as replacing willpower and mindset, which were seen as essential ingredients of a successful quit attempt:

I’d probably have to go to the doctor and ask to go for the Champix or something because as I said I’m on patches at the moment, that’s not effective. But I do know that I have to change my personal situation so that’s helpful and my mindset changes too.

*(Female, 55+, 11–20 CPD)*

Interestingly, the few participants who had used prescription medication found it effective and reported positive attitudes towards it, despite a subsequent relapse.

Yeah, I reckon that Champix, like that helped me. I slowed down so much in the first two weeks like from going to 20 a day I might have like one in the morning, one at night sort of thing and then maybe, and then a bit further on maybe just one at lunch, that’s it. Then I stopped taking it, like I sort of messed up, muddled up and yeah then I just started smoking more and smoking more and you go oh, I’m smoking again. But I think if I had have continued with it I probably I want to give it another go, so.

*(Male, 26–40, 11–20 CPD)*

As already described, guidelines for treating tobacco dependence recommend that counselling is combined with pharmacotherapy. Few participants in this study reported any personal experiences with counselling for smoking cessation. This is despite the widespread promotion of Australia’s *Quitline*: a free, government-funded telephone counselling service that can be accessed by any smoker. The number for the *Quitline* is displayed prominently on all Australian cigarette packs, and health professionals are encouraged to refer smoking patients to the *Quitline*. In addition, referral to a counselling service, which is typically the *Quitline*, is a necessary condition for doctors to prescribe subsidised NRT or prescription medications for patients. Participants reported a number of negative perceptions of the *Quitline*, including that it was “preaching”, that there was nothing *Quitline* counsellors could tell smokers that they didn’t already know, and that it was scripted and impersonal.

Again I think that is completely dependent on the person. I’m far too stubborn to ever listen to anything like that. I think it would just make it worse if someone was preaching to me, which is the way I would see it, whether it was actually like that or not.

*(Female, 26–40, 1–10 CPD)*

It was common for participants to state a preference for “personal” support from family and friends.

A less commonly discussed theme was a lack of interest in counselling. A number of participants said that they “weren’t talkers” and therefore were not inclined to use counselling to quit smoking. Even those who expressed positive views of counselling were reluctant to use it for smoking cessation. Only one person intended to use the *Quitline* on their next quit attempt, and two said that they would use generic counselling.

Participants were also questioned about their views on self-help material such as books, pamphlets, and online information. While participants had moderately positive views about self-help materials, they did not hold strong views about them. A handful of participants described specific materials that they had found useful. The framing of the message was described as being important, with some complaining about “scare-mongering” in self-help materials. Self-help materials were perceived by a few as insufficient for quitting smoking. Others were not interested in them because they did not enjoy reading.

Could be good, yeah. Depends on individual-if someone is having reading as a hobby, could be helpful. People like me who is not really into reading, yeah, could be waste of time for me.

*(Male, 26–40, 1–10 CPD)*

## 4. Discussion

We found that smokers’ attitudes towards cessation options were shaped by several factors, some of which were consistent across different methods for smoking cessation. Many participants believed that the best method for quitting would “depend on the person”. Dispositional or character-based factors were often cited when evaluating the potential of a quitting method. Unassisted quitting was seen as suitable for those with willpower, strong motivation or internal strength. The nature of an individual’s addiction to smoking was also seen as important when smokers deliberated about cessation options, as was perceived efficacy, which was typically assessed on the basis of their own experience, or that of family and friends. Negative experiences of friends and family were frequently reported, perhaps because such experiences are more salient than positive ones.

Practical factors such as cost or side effects were regarded as significant for some quitting methods. Cost was mainly mentioned as a barrier to using NRT, less often for prescription medication. Side effects were discussed frequently in relation to NRT and prescription medication. The number of side effects mentioned by study participants, particularly in relation to prescription medication, was higher than what would be expected from epidemiological evidence [35,36]. This may be because smokers misinterpret nicotine withdrawal symptoms as side effects of smoking cessation medications; or because people are more likely to discuss experiences of medication use where they have experienced side effects than those in which they have not.

Our finding that some participants did not use NRT in accordance with clinical recommendations is consistent with evidence from quantitative surveys. The latter have found that most smokers do not use NRT as directed and few use a full course of NRT as recommended [37]. This lack of adherence increases the likelihood that withdrawal symptoms will be experienced and perhaps mistaken for side effects.

The fact that not all participants were aware of the existence of prescription medications is not surprising given the lack of direct to consumer advertising of prescription medications in Australia. Additionally, since one of the indications for the prescription of smoking cessation medications in Australia is smoking more than ten cigarettes per day, it should be expected that lighter smokers will have less awareness of prescription medications for smoking cessation [5]. Research in the UK has shown that young and healthy smokers who attend their doctors are less likely to be prescribed pharmacotherapy for smoking cessation than older smokers with existing health problems [38]. While our research was not able to assess this possibility, it may be that different types of smokers are being provided with different information about their quitting options by health practitioners.

It is interesting that smokers’ concerns about side effects usually trumped efficacy, especially in the case of prescription medications. Even if smokers perceived prescription medication to be helpful, this was weighed against the risk of side effects that many decided made the potential benefits not worth the risk. The literature on risk perception in smoking shows that the distal nature of the health risks are a deterrent to quitting, particularly for young people, as they often hold optimistic beliefs about their ability to quit smoking prior to developing any smoking related health problems [39]. Any side effects from using pharmacological cessation aids for smoking cessation are more immediate, and may therefore take precedence over the longer-term health risks of smoking. This is consistent with evidence on the use of medications more generally, where the difference between the perceived necessity of a medication and concerns about its use predict poor medication adherence [16]. It is important that future research assessing smokers’ attitudes towards quitting methods take their more general views on the use of medications into account in the study design.

Beliefs about addiction were influential in our smokers discourse on smoking cessation. For example, those who believed that they were not physiologically addicted to nicotine, or who did not consider themselves to be “heavy” smokers, did not see pharmacological cessation aids as appropriate for them. These participants were more likely to hold positive views about cold turkey quitting. This is a complex topic for health practitioners to negotiate. One potential implication of this finding might be that smokers need to be educated about nicotine addiction in order to convince them that they have a physiological dependence that can be treated using medications. However, as Chapman and McKenzie [10] argue, such an approach may unintentionally devalue unassisted quitting, and produce a counterproductive effect in which smokers who are told how difficult it will be for them to quit smoking, are less inclined to try to quit. Indeed, a recent paper suggests that health practitioners “emphasize the difficulty of quitting without assistance” in order to promote uptake of medications for smoking cessation [40]. We suggest that a more sensitive, tailored approach is employed by health care practitioners. Where patients are very averse to medications, it would be counterproductive to emphasize the difficulty of quitting unassisted. Probing patients about their views on nicotine addiction and their attitudes towards medications may aid doctors in designing individualised treatment plans for patients who have tried and failed to quit cold turkey on a number of occasions.

Our study shows that smokers evaluate a given method for quitting in light of a range of alternatives and contingencies. For example, when thinking about varenicline, smokers might think that it will be effective, but believe that the side effects are not worth it. Unassisted quitting is seen as a particularly salient alternative to pharmacological cessation aids because it is free, safe and perceived by many smokers to be the most effective way to quit. This point has been neglected in the smoking cessation literature, where smokers’ views on unassisted quitting have not often been sought [9]. Males in particular preferred cold turkey quitting because they anticipated a strong sense of achievement from quitting without help. The value placed on this sense of achievement from quitting unassisted has been observed in another study [26]. It may be helpful to take this into account when designing interventions aimed specifically at men.

Our research suggests that it was common for smokers to believe that quitting cold turkey will only be effective if the smoker is “ready” to quit, and has the right “mindset”. This idea that smokers need to be ready to quit has also been prominent in smoking cessation literature and programs, thanks to the influence of the transtheoretical model of behaviour change. The transtheoretical model posits that individuals pass through a set of ordered stages in their journey to behaviour change, and that different interventions are suitable for different stages of change. For example, those in the “pre-contemplation” stages are not yet psychologically ready to change their behaviour. Interventions aimed at people in this stage are primarily informational and aim to increase desire and motivation to move smokers into the next stage–contemplating a quit attempt- rather than promoting an immediate attempt to quit. The transtheoretical model has been strongly criticized on the grounds that behaviour change is more dynamic and complex than the model assumes [41] and that unplanned, spontaneous quit attempts may be more successful than planned ones [17,42,43]. Moreover, the belief that an unqualified desire to quit is required prior to a successful quit attempt has been identified as a barrier to making quit attempt [6,44]. Our research is consistent with this view, with many smokers stating that they would quit at some precise point in the future when they were “ready”. It may be effective for stop smoking campaigns to challenge the idea that you need to achieve a “readiness to quit.” Along these lines, a recent commentary has recommended that all smokers presenting to their primary health physician should be provided with treatment, regardless of their expressed readiness to quit [6].

The results of this study are also consistent with previous qualitative research in showing that smokers consider willpower, strength, and motivation as central to successful quitting [26,27,45]. Cessation aids were not perceived as “magic bullets” for cessation. Rather, smokers emphasised that willpower and personal choice were necessary, even when cessation aids were used. This discourse of “willpower” has long been central to lay accounts of smoking. It aligns with Western cultural values of free choice and individual strength. It is a view that has been heavily promoted by the tobacco industry to argue for fewer government interventions to prevent or discourage smoking [46].

Even with the increasing biomedicalisation of smoking cessation, it seems highly unlikely that the discourse of willpower will disappear from public discourse on smoking. Therefore, incorporating beliefs about willpower into smoking cessation campaigns and clinical interactions may be of value. For example, messages that tell people who are using pharmacological cessation that willpower is still required may allow successful quitters to attain the sense of achievement that was valued by some in our study. It also provides more realistic expectations about the efficacy of current pharmacological options. Relatedly, only a small minority of participants believed that cessation aids would be necessary and sufficient to quit smoking. This finding should allay the concerns of those who fear that the medicalisation of smoking cessation will creates a sense of fatalism and decrease smokers’ sense of control over their smoking.

The negative views of the *Quitline* expressed by participants are consistent with evidence of low uptake of counselling in general [47,48]. This may be of concern, given that counselling is required in conjunction with the prescription of pharmacological cessation aids in clinical practice guidelines. Despite many acknowledging the psychological and behavioural aspects of smoking, few participants expressed an interest in counselling and only one participant intended to use the recommended combination of pharmacotherapy and counselling for their next quit attempt.

One limitation of this study was that participants were asked about their views of unassisted quitting using the prompt “cold turkey.” Cold turkey is generally taken to mean quitting suddenly, rather than gradually cutting down on the number of cigarettes. Cutting down is a method of quitting that is commonly used by smokers but we did not directly ask about it. Future research in this area should ask about cutting down separately from quitting “cold turkey”, or more clearly describe methods of unassisted quitting before questioning participants.

Lastly, although nicotine replacement products are widely advertised in Australia, there is no direct to consumer advertising of prescription medications. It would be useful to examine attitudes in countries where direct to consumer advertising for prescription stop-smoking medications is permitted (e.g., New Zealand, USA) to see if advertising influences smokers’ attitudes towards prescription cessation aids or unassisted quitting.

## 5. Conclusions

It should be noted that this was qualitative research and no inferences about the prevalence of these beliefs in the larger population of smokers can be drawn. However, these findings provide an insight into the range of factors that smokers consider when evaluating quitting methods. This information is useful to inform future work in this area. Specifically, smokers’ judgments about which methods to use for smoking cessation are not simply based on perceived safety and efficacy. They reflect their ideas about the nature of their addiction, how well a given method suits their perceived situation and personality, and their own and others’ experiences with the method. Their views about different methods are often not independent. For example, views about NRT are shaped by very positive attitudes towards quitting cold turkey. Looking at attitudes towards assisted or unassisted quitting in isolation may provide incomplete information on quitting preferences. It is therefore important that the above-mentioned factors are considered when conducting research into treatment preferences for smoking cessation. Smokers’ views should be compared across different quitting methods and at the very least, include quitting unassisted as a comparator.

## Supplementary Files

Supplementary File 1
