# Supplementary material for: Why Don’t Smokers Want Help to Quit? A Qualitative Study of Smokers’ Attitudes towards Assisted vs. Unassisted Quitting
Source: Int J Environ Res Public Health. 2015 Jun 10;12(6):6591–607. doi: 10.3390/ijerph120606591 (PMC4483718; doi:10.3390/ijerph120606591)
Supplement: Supplementary File 1 [file ijerph-12-06591-s001.pdf]

Supplementary Information

Why Don't Smokers Want Help to Quit? A Qualitative Study of Smokers' Attitudes Towards Assisted *vs.* Unassisted Quitting

---

Smokers' Understanding of Nicotine Addiction—Survey

The answers to these questions will be used to make sure that we have spoken with a range of different people with different experiences. Please do not write your name on the form.

ID Number:

1. Are you? (tick one)

   Male ☐ Female ☐

   What is your age? ………………………. (years)
2. What is your employment status? (tick as many as apply)

   ☐ Working full time
   ☐ Working part-time
   ☐ Studying full-time
   ☐ Studying part-time
   ☐ Doing home duties
   ☐ Unemployed
   ☐ Retired
3. What is your highest level of education? (tick one)

   ☐ Did not complete senior secondary school
   ☐ Completed senior secondary school (e.g. Higher School Certificate)
   ☐ Trade/Apprenticeship (e.g. hairdresser, chef)
   ☐ Certificate (Level III or higher) or diploma (e.g. child care, technician)
   ☐ University degree (e.g. Bachelor)
   ☐ Postgraduate university degree (e.g. Graduate Diploma, Masters, PhD)
4. What is the postcode of your usual residence? ………………
5. What is your occupation? (if applicable)

   ………………………………………………………….
6. How long after waking do you wait before having your first cigarette (in mins?)

   ☐ 0–5 minutes
   ☐ 6–30 minutes
   ☐ 31–60 minutes
   ☐ 61 minutes or more
7. How many cigarettes do you smoke per day at present?

   ☐ 1–10
   ☐ 11–20
   ☐ 21–30
   ☐ 31 or more

8. How often do you get strong urges to smoke?
   - ☐ Never
   - ☐ Less than daily
   - ☐ Daily
   - ☐ Several times a day
   - ☐ Hourly or more often
   - ☐ Not applicable
   - ☐ Don't know
9. Quit attempts

   a. Have you ever tried to quit smoking?
   - ☐ Yes
   - ☐ No

   b. What is the longest you have gone without smoking in the last 5 years?
   - ☐ Have not stopped
   - ☐ Less than a day
   - ☐ A day
   - ☐ Up to a week
   - ☐ Up to 4 weeks
   - ☐ Up to 3 months
   - ☐ Up to 6 months
   - ☐ Up to 12 months
   - ☐ Longer than 12 months

   c. Have you used any of the following to help you to quit smoking?
   - ☐ Discussed smoking and health at home
   - ☐ Rung the "QUIT" line
   - ☐ Asked your doctor to help you stop smoking
   - ☐ Used nicotine gum, nicotine patch, or nicotine inhaler
   - ☐ Used a smoking cessation pill (e.g. Zyban, Champix)
   - ☐ Brought a product other than nicotine patch, gum or pill to help you quit
   - ☐ Read "How to Quit" literature
   - ☐ Used the internet to help you quit
   - ☐ Done something else to help you quit? Please specify……………………………………
   - ☐ None of the above

10. Are you planning to give up smoking?
    - ☐ Yes, within 30 days
    - ☐ Yes, after 30 days, but within the next 3 months
    - ☐ Yes, but not within the next 3 months

☐ No, I am not planning to give up

Thank you for your participation in this study.

© 2015 by the authors; licensee MDPI, Basel, Switzerland. This article is an open access article distributed under the terms and conditions of the Creative Commons Attribution license (http://creativecommons.org/licenses/by/4.0/).
